# Machine Perfusion and Bioengineering Strategies in Transplantation—Beyond the Emerging Concepts

**DOI:** 10.3389/ti.2024.13215

**Published:** 2024-08-29

**Authors:** Anna Niroomand, George Emilian Nita, Sandra Lindstedt

**Affiliations:** ^1^ Department of Clinical Sciences, Lund University, Lund, Sweden; ^2^ Lund Stem Cell Center, Lund University, Lund, Sweden; ^3^ Department of Cardiothoracic Surgery and Transplantation, Skåne University Hospital, Lund, Sweden; ^4^ Department of Transplantation Surgery, Liverpool University Hospitals NHS Foundation Trust, Liverpool, United Kingdom; ^5^ Institute of Systems, Molecular and Integrative Biology, University of Liverpool, Liverpool, United Kingdom; ^6^ Division of Transplantation Surgery, Karolinska University Hospital, Stockholm, Sweden; ^7^ Wallenberg Center for Molecular Medicine, Lund University, Lund, Sweden

**Keywords:** transplantation, machine perfusion, bioengineering, gene therapy, cross circulation

## Abstract

Solid organ transplantation has progressed rapidly over the decades from the first experimental procedures to its role in the modern era as an established treatment for end-stage organ disease. Solid organ transplantation including liver, kidney, pancreas, heart, and lung transplantation, is the definitive option for many patients, but despite the advances that have been made, there are still significant challenges in meeting the demand for viable donor grafts. Furthermore, post-operatively, the recipient faces several hurdles, including poor early outcomes like primary graft dysfunction and acute and chronic forms of graft rejection. In an effort to address these issues, innovations in organ engineering and treatment have been developed. This review covers efforts made to expand the donor pool including bioengineering techniques and the use of *ex vivo* graft perfusion. It also covers modifications and treatments that have been trialed, in addition to research efforts in both abdominal organs and thoracic organs. Overall, this article discusses recent innovations in machine perfusion and organ bioengineering with the aim of improving and increasing the quality of donor organs.

## Introduction

Since the start of successful kidney transplantation in the 1950s followed by other solid organs, transplantation has taken on a steadfast role in medicine as the definitive treatment to improve the quality of life and prolong the survival of patients with end-stage organ disease [1–6]. In recent decades, major advances in surgical technique, and pre-and post-operative management have allowed for the prolonged survival of recipients. As the number and availability of transplants have increased, the imbalance between organ supply and demand has grown to unprecedented levels [7]. In 2024, the Organ Procurement and Transplantation Data showed over 113,000 patients waiting for a transplant on the US all-organ waiting list [8], while the NHS Blood and Transplant reported over 7,000 [9] potential recipients in the UK alone and Eurotransplant listed over 13,000 patients currently active in eight European countries [10].

To address this disparity, an increase in the number of available donor organs is needed, which could theoretically be accomplished through a variety of means, including increased organ donation, procurement, and recuperation of available grafts. Restoration and regeneration of damaged donor organs is a particularly promising path for recovering a greater number of donor grafts, considering the high percentage that are currently discarded.

Grafts considered for transplantation may be rejected for several reasons. Factors such as the age of the donor, the cause of death, and the resulting sequelae of damage to the organ are often the primary reasons for rejecting the organ for transplantation. In the evaluation of donor lungs, for example, injury from trauma, infection, and aspiration may lead to acute lung injury (ALI) and the resulting functional impairment may preclude the organ from being chosen for transplantation [11, 12].

Many of these once-rejected organs are now being reconsidered in an effort to expand the donor pool. For example, the limits of what constitutes an acceptable organ have been raised with the introduction of expanded criteria for donors, donors at increased risk, and donors with acute kidney injury [13–17]. Despite these efforts, there are still organs being discarded that could theoretically be recovered for use via advanced techniques. The utilization of bioengineered organs is an expanding and promising approach and the creation of functional bioengineered organs could contribute to an immunosuppression-free state [18]. This article reviews the use of machine perfusion in several organs, exploring both the clinical application of this technology and the bioengineering techniques leveraged to increase the quality of donor organs.

## Machine Perfusion

Circulatory support of donor organs following organ retrieval has been developed for several solid organs, with the advantage of such *ex vivo* platforms being the ability to evaluate the functionality of organs, prolong the preservation time between explant and implant, and apply recovery therapies to the target organ.

Since Alexis Carrel and Charles Lindbergh first described *ex vivo* machine perfusion in 1935, reporting the successful perfusion of the feline thyroid gland [19], significant efforts have been made to assess organ function and prolong preservation. In the 1970s animal models of the lung [20] and Hardesty and Griffith’s publication on autoperfusion of combined heart and lung transplants were highlighted [21]. Various liver-assisting therapies were researched during the 1960s and 1970s (cross-circulation, hemodilution and plasmapheresis), but they were abandoned with the development of orthotopic liver transplantation and understanding of the possible immunologic implications [22]. Animal models utilizing cold perfusion, oxygenated blood or plasma for kidney [23–25] and pancreas preservation continued to develop in the early 1980s [26, 27]. In Lund, Sweden, *ex vivo* lung perfusion (EVLP) was brought back by Steen et al. with the first marginally viable lungs transplanted following reconditioning [28–30]. This EVLP model was further developed with the establishment of protocols for prolonged evaluation by the Toronto group [31], which led to increasing and expanding clinical application.

At their core, machine perfusion platforms consist of an organ chamber in which perfusate flows through the graft using a pump (either centrifugal or roller) via a circuit equipped with a temperature control system and an oxygenation method. In the case of lung machine perfusion called EVLP, the organ is additionally ventilated with mechanical ventilation at settings determined by the underlying protocol. In general, the flow of perfusate allows for the delivery of oxygen and nutrients as well as the removal of waste and toxins, while also providing the opportunity to deliver therapeutic agents directly to the target. Leukocyte filters may also be incorporated into the circuit, although the efficacy of such an addition is under debate [32].

### Lung Perfusion

In EVLP, the organ is both perfused and ventilated, which allows for additional graft monitoring both through analysis of the perfusate as well as through bronchoscopy and bronchoalveolar lavage fluid collection. An important note about the circuit specific to the lung is the need to deoxygenate the perfusate to assess the organ’s functionality, with the ratio of arterial oxygenation to a fraction of inspired air (PaO_2_/FiO_2_) being an important clinical measure.

The two most commonly used commercially available platforms for performing EVLP are the XPS™ system made by XVIVO [33] and the portable Organ Care System™ (OCS) produced by Transmedics [34]. Among these platforms, there are also three commonly referenced protocols, including the Lund protocol [28], the Toronto protocol [35], and the OCS protocol [36]. These differ in several parameters including the target perfusate flow (often calculated based on cardiac output), whether the left atrium of the donor lung graft is open or closed, and the composition of the perfusate. Regarding the perfusate, the differences lie in the underlying base solution (STEEN vs. OCS), and the use of red blood cells vs. acellular perfusate (Lund vs. Toronto). The debate over the use of cellular versus acellular perfusate continues, with some reports finding that cellular perfusate achieves results approaching clinical standards and superior lung preservation as well as a reduced incidence of lung edema and improved compliance in a porcine model. Despite these preclinical findings, human studies using EVLP have focused on acellular perfusates with favorable results.

Pioneering investigations have established the efficacy and utility of these systems in clinical applications. Starting in 2011, the Toronto group demonstrated in the HELP trial that transplantation with high-risk donor lungs maintained on 4 h of EVLP could produce comparable results to conventional lungs using their EVLP protocol [31]. The NOVEL trial which followed a few years later in 2014 extended these findings to a multicenter trial in 6 US centers with expanded criteria donors compared to conventional lungs and again encouraged the use of EVLP [37]. In the 2016 DEVELOP-UK trial, the EVLP observational cohort was terminated early, with lower survival rates, higher rates of ECMO and early grade 3 primary graft dysfunction (PGD) in the EVLP arm which utilized lungs not suitable for standard transplantation [38]. Other reports subsequently have emerged that provide contrasting support for the continued use of EVLP, including the 2018 INSPIRE and 2019 EXPAND trials using the OCS system [36, 39]. The INSPIRE trial compared standard criteria lung transplants maintained with EVLP versus cold storage, with findings of reduced PGD grade 3 at 72 h in the EVLP arm and meeting the primary safety endpoints. The EXPAND trial was then expanded to include extended criteria donors with primary endpoints of 30-day survival and absence of grade 3 PGD. Although the performance goals of the study were not met, the study group concluded that the use of EVLP allowed the transplantation of organs that would otherwise have been rejected. Similarly, supportive data for EVLP from a single-center analysis in Vienna was published in 2017 reporting that in their use of EVLP in standard donor lungs, EVLP use provided evidence of functional results and operative outcomes comparable to standard preservation, with the cited benefit of safely extending preservation time [40].

Clinical EVLP systems have since evolved to be executed not only by a transplant service’s own team (referred to as local in-house EVLP) but also to be performed at an outside but centralized instutution (sometimes called a centralized lung evaluation system). This centralized version has recently gained traction due to the development of Lung Bioengineering’s dedicated facility in which remote EVLP was performed for seven different United States-based lung centers [41]. Such centralized EVLP may grow in popularity with the advent of the first European centralized facility coordinating EVLP between Sweden and Denmark [42].

Within these applications, the details of optimal parameters are still being discussed and guidelines for optimal execution need to be established. Ventilation methods are an additional point of interest, particularly because of the known effects of harmful ventilation causing ventilator-induced lung injury. Because excessive pressure (barotrauma), alveolar collapse (atelectrauma), and volume (volutrauma) can induce iatrogenic damage to the graft, attention to the method of ventilation is critical. The stress index which represents the rate of change in compliance during tidal inflation has been proposed as a means of assessing ventilation in EVLP, with high indices corresponding to hyperinflation and low indices corresponding to recruitment-derecruitment [43, 44]. Ventilation outside of the stress index window has been reported to correlate with cytokine-driven inflammation and longer time on mechanical ventilation, intensive care unit (ICU) stays, and hospital stays [45]. Using an auxiliary device, exhaled breath particles can also be collected from the mechanical ventilation setup on EVLP, which has shown that greater numbers of particles are correlated with higher tidal volumes and volume-controlled ventilation [46]. Higher particle flow rates using this methodology have been correlated with lung injury [47, 48] and with primary graft dysfunction in the transplant setting [49]. Other studies have investigated airway pressure release ventilation compared to conventional volume-controlled ventilation in a porcine model to try an open lung approach to ventilation, which reduced edema during EVLP and improved PaO_2_/FiO_2_ ratios following 4 h of reperfusion after left lung transplantation [50]. Negative pressure ventilation was trialed in another porcine experiment with 12 h of *ex vivo* perfusion and found to have lower levels of pro-inflammatory TNF-α, IL-6 and IL-8 along with less edema during EVLP [51].

### Heart Perfusion

Similar to the lung, the perfusion circuit of the *ex vivo* machine can be altered to accommodate the specific needs of the heart, including wires that allow for defibrillation and pacing. Currently, the two systems in place for *ex vivo* heart perfusion (EVHP) are the Heart Box™ (XVIVO) and the portable Organ Care System (OCS) Heart™ (TransMedics). The main difference between these systems is the use of normothermic perfusion in the OCS Heart versus hypothermic preservation at 8°C in the Heart Box. The reported benefits of EVHP in these systems are notable not only for the clinical results obtained, but also for the system’s ability to assess hidden cardiac pathologies [52] and to find specific and sensitive markers of outcome [53].

In the 2014 trial of normothermic heart perfusion, standard criteria donors were compared against conventional preservation techniques, looking at the survival of the recipients over a 2-year period [54]. During this time, survival rates were comparable in the early 30-day post-transplant period, and surpassed cold static storage at one and 2 years along with a reduction (although not to a statistically significant degree) in primary graft dysfunction. In the European-based PROTECT trial [55], EVHP was evaluated for safety and was followed several years later by the PROCEED II trial, a prospective, randomized non-inferiority trial in 10 different transplant centers [56]. Short-term clinical outcomes were again comparable between groups at 30 days and in a one-center follow-up to the 2-year mark, there was again no significant difference in survival despite significantly longer ischemic times in the EVHP group [57]. Separately from the EXPAND lung trial, an EXPAND heart preservation trial examined the utility of the system for extended criteria donors, with primary endpoints of survival at 30 days and absence of PGD at 24-h post-transplant [58]. In this study, the perfusion was not compared to standard preservation techniques, but did show high utilization of donor grafts, low rates of PGD, and high survival rates. To further characterize its use in unfavorable donors, Sáez et al. placed extended criteria donor hearts on machine perfusion, describing their single-center experience using the system and reporting improved short-term outcomes, although this was not directly compared to standard criteria [59]. In the same group, patients who were bridged to transplantation using left ventricular assist devices were compared to those whose donor organ was preserved with EVHP versus standard cold storage [60]. Recipients whose grafts had received machine perfusion had lower rates of mechanical circulatory support and decreased need for blood transfusion with longer out-of-body graft time, although ultimately there was no significant difference in overall survival [60]. Another examination of extended criteria transplants supported by EVHP compared to conventional storage showed that there was an effective increase in the number of transplants possible given the recovery of organs using the system, and that the survival of the donor hearts was comparable in terms of both hospital length of stay and survival [61]. A long-term follow-up report of normothermic EVHP from the Berlin group followed recipients 4 years post-transplantation and showed high survival rates, supporting the use of the platform [62]. Other notable smaller studies include the case report of prolonged out-of-body time of 10 h using machine perfusion [63], as well as a case series from St. Vincent’s in Australia demonstrating distant procurements on donation after cardiac death (DCD) with favorable results [64]. This group also demonstrated favorable lactate profiles during EVHP in their porcine DCD model, in which they also reported that following orthotopic transplantation, only those previously placed on EVHP were able to be weaned from cardiopulmonary bypass support [65].

The hypothermic alternative to EVHP has been trialed in both porcine and human cardiac transplantation. In a study of porcine DCD procurement, following normothermic regional perfusion, cold storage was compared to hypothermic machine perfusion, finding that contractility was only found with hypothermic EVHP along with a lower need for inotropic support and signs of myocardial damage [66]. A trial of hypothermic EVHP was run by the Australian St. Vincent’s group, with a demonstration of 13 transplants, in which one patient required post-operative VA-ECMO and no postoperative mortality was observed at 30 days [67]. In the first prospective trial of hypothermic EVHP by the Lund group, standard criteria donors were compared in both modalities, showing improved mortality and cardiac-related adverse events in the machine preservation arm [68]. The HOPE trial followed with a multicenter investigation of the system in donor hearts with both short and long preservation times, up to 8 h and 47 min. When compared to comparator data retrieved from the International Society of Heart and Lung Transplantation (ISHLT), the EVHP group demonstrated improved 30-day survival and low rates of PGD development [69].

### Liver, Kidney, and Pancreas Perfusion

The machine perfusion landscape in liver, kidney, and pancreas transplantation has been similar to that of thoracic organ transplantation. Attempts to improve donor organ viability, functional assessment, and overall graft and patient outcomes led to the rapid expansion of *ex vivo* hypo- and normothermic machine perfusion (HMP, NMP) and *in vivo* normothermic regional perfusion (NRP).

The concept of NMP was developed in the 1960s when it was initially studied for kidney preservation. Its development over the years has expanded its application to the liver [70], significantly improving outcomes by lowering graft injury, and discarded organs and increasing the mean preservation time compared to conventional static cold storage (SCS) techniques. Similarly, HMP was conceptualized in the 1960s. Significant advances in HMP technology were made in the 1990s through the pioneering work of Belzer and Southard [71], who developed the University of Wisconsin (UW) solution for the preservation of the liver, kidney, and pancreas.

A systematic review and meta-analysis by Jakubauskas et al. compared outcomes between NMP and HMP in liver transplantation versus static cold storage (SCS). Their study demonstrated the role of machine perfusion in reducing graft injury and decreasing the rate of liver graft rejection, early allograft dysfunction (EAD) and non-anastomotic biliary strictures compared to SCS [72].

Machine perfusion in kidney transplantation showed a similar significant improvement in graft outcomes. The international randomized controlled trial by Moers et al. demonstrated a decrease in delayed graft function and an improvement in graft survival rates when utilizing HMP as opposed to SCS alone [73].

*Ex vivo* machine perfusion has also been utilized in the field of solid pancreas transplantation for quality assessment of discarded organs [74] and to improve preservation capacity, both the ability to resuscitate organs and to reduce delayed graft function [75].

## Organ Engineering on Machine Perfusion

As previously mentioned, the discrepancy between the availability of donor organs and the number of potential recipients waiting for a graft demands the development of novel therapies and treatments to bridge this gap. While *ex vivo* perfusion itself can help to evaluate marginal organs that would otherwise be discarded or facilitate the transplantation of more distantly procured grafts, there are many rejected damaged donor organs that could be retrieved. The addition of different therapies to the machine perfusion circuit presents an opportunity to recover a greater number of grafts. Advantages include highly specific temporal and spatial control of treatment delivery along with the avoidance of potential systemic effects.

### Gene Therapy

Following the adoption of viral-based vectors, gene therapy has been pursued for its potential in immunomodulation and mitigation of the inflammatory response often seen in the events surrounding transplantation.

In cardiac transplantation, early rat models using adenovirus vectors carrying LacZ demonstrated that transfection could occur with minimal effect on graft survival, but the results were tempered by short gene expression lasting only weeks [76, 77]. Subsequent efforts have demonstrated that the hypothermic *ex vivo* platform can be a reliable conduit for vector delivery, with further development of adenovirus vectors of LacZ in both porcine and rat models and a need for continuous perfusion beyond single injections [78–80]. Further testing in rabbit models of normothermic EVHP demonstrated transfection using similar vector-gene combinations [81, 82]. Notably, the Duke group produced a porcine model in which luciferase was transfected and investigated the components of the platform that promoted or hindered successful transfection [83] in a 5 day post-transplantation model. This group additionally reported on a recombinant adeno-associated virus carrying luciferase that showed high efficiency and expression for 30 days without off-target effects or signs of rejection [84].

Within the heart, targets mainly aimed at immunomodulation have been identified and successfully delivered in animal models, including constructions of interleukin-10 or transforming growth factor-beta 1 (TGF-β1), in which transplanted grafts have shown higher vector concentrations as well as prolonged graft survival and inhibition of allograft rejection [85, 86]. Other vectors have included the delivery of a CTLA4 gene with a demonstration of localized immunosuppression in rats [87] and liposome-based delivery of IL-4 and IL-10 to rabbits with significantly increased survival and reduced rejection [88].

In the lung, gene therapy has also gained momentum for its potential for immune regulation. An important decision point is the route of administration: intravenous delivery is theoretically better at targeting the endothelium while the endotracheal route can target the epithelium. Several studies have investigated these routes of delivery, with the endotracheal route often being preferred [89, 90]. In earlier studies performed on rat transplantation by the Toronto group, the feasibility of gene transfection was explored using six and 12 hour *ex vivo* transfusions, although importantly this was done in cold preservation prior to the advent of modern EVLP [91]. In further developments of the model, human IL-10 within this adenovirus vector was studied, again with six or 12 h of *ex vivo* incubation with a reported improvement in post-transplant organ function [92]. The IL-10-containing vector was further examined in porcine and human lungs, now placed on modern EVLP systems, with endotracheal administration of treatment [93]. In the porcine model, following transplantation with a 4 h follow-up, cytokines IL-6 and IL-1β were significantly decreased in the transfected group and the rejected human organs, there was no observed deterioration in function on EVLP. The group compared delivery of the viral vector using an *ex vivo* model with an *in vivo* model, and in both cases transplanted the lung to assess post-transplant function. The *ex vivo* group showed improved lung function compared to the *in vivo* group, with the authors hypothesizing that this methodology led to less inflammation associated with vector delivery to explain the improved function [94]. In a prolonged 7-day survival model, the same therapy was applied, demonstrating continued IL-10 expression through day 7 in addition to a lack of systemic toxicity, and improved lung function on day 7 in the treated group compared to the control conditions [95]. Of note, transfection with an IL-10-carrying vector has been shown in rodent models of transplantation without the use of EVLP and has shown decreased rates of histologically scored acute rejection [96], lower levels of inducible nitric oxide synthase [97], as well as improved gas exchange and decreased IL-2 expression [98].

Gene therapy in the lung has also been pursued with other targets, notably with the delivery of small interfering double-stranded RNA (siRNA) via lentiviral vectors targeting the class-II-trans-activator and β2-microglobulin, which ultimately reduced MHC-1 and II transcription in lung endothelial cells in a porcine model [99]. This mechanism leveraged by Figueiredo et al. allows for the evasion of the transfected cells from immune system activation, a valuable attribute when transplanting a donor organ into a recipient.

Gene therapy in kidney transplantation offers a novel approach to addressing common post-transplant complications such as rejection, ischemia-reperfusion injury (IRI), and the nephrotoxic effects of immunosuppressive drugs. By targeting the genetic foundations of these issues, gene therapy has the potential to significantly improve transplant outcomes. Bogacz et al. investigated the correlation between tacrolimus dose and genetic variation for IL-10 and its effect on therapeutic outcomes in kidney transplantation patients, revealing the potential influence of IL-10 polymorphism on immunosuppressive drug dosage and the risk of acute graft rejection [100]. In cold-IRI mouse models, the anti‐IL‐2 immune complex was found to attenuate ischemia‐reperfusion injury (IRI) after kidney transplantation by increasing renal regulatory T cells [101].

In the pancreas, gene therapy can introduce encoding for immunomodulatory proteins (e.g., interleukin-10, TGF-beta) to create a local immunosuppressive environment. An example is the role of the VEGF gene in modifying isolated pancreatic islets for transplantation. VEGF has anti-apoptotic and angiogenic effects that are critical for islets under hypoxic stress during transplantation. Studies reveal that islets modified with the VEGF gene display have improved viability and more effective glucose regulation post-transplantation compared to non-modified islets [102].

Gene therapy in liver models in the context of transplantation remains limited [103–105]. Lorvellec et al. and the UCL group designed an *in vitro* whole liver [106].The “Bioreactor grown Artificial Liver Model” (BALM) concept is a custom-designed 3D culture of human induced pluripotent stem cell-derived hepatocyte-like cells (hiHEPs) on a decellularized mouse liver scaffold. Adenoviral and lentiviral vectors introduced by intravascular injection have demonstrated that BALM has the potential to serve as a substitute for some *in vitro* and *in vivo* therapeutic testing methods.

It is of course important to consider the effects of the therapy delivered to the target organ and whether there are any unintended consequences. Potential risks of gene therapy include the possibility of certain types of cancer in the long term, including the suspension of Bluebird Bio’s clinical trial of LentiGlobin gene therapy for sickle cell disease due to cancer diagnoses in two recipients 5 years after the treatment [107]. Although the lentiviral vector may not have been the direct cause of cancer, there are still concerns about how well we really understand the long-term sequelae of the treatments we deliver. This highlights the need for studies that not only look at the immediate consequences of gene therapy in transplantation, but also at what might occur to treated organs years down the line. Other hypothetical concerns include how gene therapy could potentially activate the immune system, which would be of great concern in immunosuppressed transplant recipients, and whether there are off-target effects, which would ideally be mitigated by the use of therapies in an *ex vivo* setting where the therapy was applied directly to the target organ. Of course, it is critical to note that gene therapy is not a panacea for all transplantation troubles. Often, the disease processes that emerge are complicated and multifactorial and it is unlikely that any one target identified by gene therapy will provide a universal answer to transplantation-related complications. It is likely that multimodal therapies will be needed in order to truly optimize grafts for transplantation.

### Mesenchymal Stromal Cells (MSCs)

Cell therapy has emerged as a form of treatment for damage owing to ischemia-reperfusion injury (IRI) and acute lung injury due to the properties exhibited by mesenchymal stromal cells (MSCs). This cell population has been shown to act on inflammatory pathways and play a role in immunomodulation, with multi-targeted effects and has been studied for its paracrine effects leading to tissue regeneration. Bone marrow-derived MSCs have been linked to increased levels of IL-10 to promote anti-inflammation [108] as well as the secretion of immunomodulatory growth factors [109]. A further advantage is the lack of co-stimulatory CD40, CD40L, CD80, and CD86 which confer a lack of immunogenicity to MSCs [110], making them a suitable therapy for immunosuppressed and sensitive recipients.

In lung transplantation, EVLP has provided a platform for MSC delivery. At the University of California, San Francisco, cells applied during *ex vivo* perfusion were shown to restore endothelial permeability in damaged lobes of human lungs and improve alveolar fluid clearance [111]. Improved alveolar fluid clearance was also observed with the treatment of cell culture medium conditioned with MSCs without the cells themselves. Further studies of rejected human lungs within this group also showed improvement in alveolar fluid clearance with 5 × 10^6^ cells instilled into the lungs on EVLP [112]. In studies of cells isolated from human umbilical cord tissue by the Toronto group, administration of MSCs aimed at ameliorating prolonged cold ischemia in previously healthy donor lungs was performed first with the intent of determining ideal cell concentrations following a comparison of intrabronchial vs. intravascular routes of administration [113]. As a result, intravascular administration was found to be superior given the higher airway pressures associated with intrabronchial doses, as well as the identification of a suitable dose equivalent to 5 × 10^6^ cells per kg in the porcine model. In a longer cold storage experiment with 24 h of cold ischemia followed by 12 h of EVLP, MSCs showed decreased apoptosis and reduced pulmonary edema after transplantation consisting of 4 h of reperfusion [114]. In an effort to leverage the immunomodulatory effects of MSCs, cells were engineered to produce anti-inflammatory IL-10 and were given to human lungs rejected for transplantation under 12 h of EVLP [115]. In this study, there were no significant differences in pulmonary vascular resistance, oxygenation capacity, and compliance compared to control lungs, which was attributed to an acidic lung microenvironment and heterogeneity of injury across human organs. Umbilical cord-derived MSCs were also examined during rat EVLP by Pacienza et al., indicating that intravascular infusion showed better lung compliance in addition to a reduction in neutrophil-related markers, supporting an anti-inflammatory role of the cell treatment [116].

In the heart, *ex vivo* perfusion has only been studied with the application of MSC-conditioned medium, rather than the stromal cells themselves. In an investigation of hypothermic perfusion in rats, a conditioned medium was associated with the downregulation of proinflammatory cytokines following transplantation and was hypothesized to protect against myocardial IRI [117]. In a later study by the same group, the conditioned medium was tested in brain-dead donors, showing improved systolic function after preservation and reduced cell apoptosis [118]. In normothermic EVHP, the conditioned medium as studied by the Guangzhou group showed that oxidative stress, inflammation, and apoptosis were mediated by the treatment and that warm ischemic injury was improved [119]. When the same group transplanted the conditioned hearts, spontaneous contraction and left ventricular systolic diameter improved in the treated group and lower levels of plasma cytokines were detected [120]. Compared to whole-cell treatment, these studies offer an opportunity to explore the conditioned medium as a therapy in and of itself, which may have advantages over MSC administration in terms of both time and cost savings. Interestingly, MSCs have been studied in the treatment of ischemic and non-ischemic cardiomyopathy and have even been labeled as cardioprotective, implying an avenue for further investigation in the transplantation setting [121–127].

In the kidney, MSC therapy has also been studied for its potential to repair damage from ischemia, which was tested in a human kidney model of *ex vivo* perfusion. MSC treatment showed reduced inflammatory cytokines and increased adenosine triphosphate and growth factors [128]. In a rat model, kidney grafts were treated with MSCs or with extracellular vesicles released by the MSCs, both of which were associated with improved metabolism and ion transport and Gregorini et al concluded that both forms of treatment protected the kidneys from reperfusion damage [129]. In later work by Pool et al, porcine kidneys were treated with MSCs during normothermic machine perfusion and labeling of bone marrow-derived MSCs showed that the cells rapidly decreased in the circulating perfusate over time and that a small portion was intact and appeared within the glomeruli [130]. The authors noted that the study demonstrated that the cells can reach and reside in the kidney, but that the infusion rate must be high enough to visualize within the capillaries. The group then also reported that with normothermic perfusion, MSC treatment at these sufficiently high cell infusion rates reduced injury markers and induced the release of immunomodulatory cytokines during ischemia-reperfusion injury [131]. MSC treatment was further followed in a transplantation model by Lohmann et al. with the finding that while MSC treatment was safe and well tolerated within their autotransplant cohort, there were no significant effects of the therapy within the 14 days post-transplantation [132]. An important question within this line of work is the dose tolerance of the kidneys to large amounts of MSCs, which was investigated by the same group through their porcine model comparing doses of ten million and one hundred million MSCs injected into the renal artery [133]. The lower dose was well tolerated, while the high dose resulted in tissue inflammation and glomerular and tubular damage. A balance must be struck between administering doses of cell therapy that are both effective and not so high as to cause damage themselves.

Extending the investigation of MSC therapy to the liver, several studies have examined the efficacy of the therapy within this solid organ. In a rat model, MSCs were administered during machine perfusion following donation after cardiac death, with treatment correlating with improved bile production and histologic findings [134]. MSC treatment was shown by another group to ameliorate injury due to oxidative stress and increase mitochondrial function within their rat liver cohort [135], which was further extended in other studies to demonstrate improved histopathology, hepatocyte ultrastructure, and microcirculation-related indices [135, 136]. In a porcine model, MSCs were shown to be retained in a machine-perfused liver with the reported continuation of their paracrine activity via increased human-specific IL-6 and IL-8 levels in porcine blood [137].

### Cross-Circulation

Although the system does not employ a machine, the perfusion method of cross-circulation is noteworthy for its novelty in the development of methods to regenerate damaged donor lungs and livers. Reported advantages of cross-circulation over machine perfusion include longer recovery times and an increased duration of extracorporeal support [138]. An early report of cross-circulation in the lung appeared under the work of Dr. Joel Cooper who reported cross-circulating aspirin-treated sheep with non-treated sheep to examine the mixing of the circulating blood volumes and to study the circulating elements [139]. More recent work has emerged to establish a cross-circulation platform as a means to rehabilitate damaged donor lungs. The Bacchetta group first reported the use of cross-circulation to achieve prolonged perfusion times in excess of 36 h, followed by a porcine study in which lungs damaged by the aspiration of gastric contents were then reconditioned using the platform [138, 140]. To demonstrate the system’s ability to provide prolonged support, porcine lungs were additionally maintained in cross-circulation for 4 days, with preserved lung function and decreased levels of several cytokines, including IL-1β, IL-8, and IL-12 among others [141]. Gastric aspiration-damaged recipients were also shown to help recover donor lungs subjected to prolonged cold storage in a model that leveraged injury in both the donor and recipient within cross-circulation [142]. In a step closer to clinical application, rejected human lungs were added to the xenogeneic circuit to show functional and histological recovery under the platform [143, 144] with 24 h of support. There are of course concerns about the immunogenic response that might follow when exposing human lungs to a xenogeneic recipient. Wu et al reported that in their established cross-circulation model, porcine immune cells and immunoglobulins can be found infiltrating human donor lung tissue after 24 h of xenogeneic perfusion [145]. Similarly, in an investigation by Glorion et al., immune cells from the porcine host were found in lung cell suspensions, in bronchoalveolar lavage fluid, and in histological examination of lung tissue [146]. The group also posited that cross-circulation could act as a platform to investigate the immune processes that follow transplantation without carrying out full transplantation and could serve as a means to assess anti-inflammatory and immunomodulatory drugs based on the response of the porcine host.

Cross-circulation has also been investigated in porcine livers, as early as 2001 when a group from Kyoto studied porcine liver perfusion with baboon cross-circulation [147]. The group found microthrombi formation in the perfused porcine livers and macroscopic hemolysis in 3 out of 5 cases with a maximum duration of 6 h of cross-circulation. In a publication from the same year, Nara et al. in Hirosaki, Japan cross-circulated hepatectomized dogs with donor pigs to test a semipermeable membrane in their model of double filtration plasmapheresis cross-circulation [148]. This model was further explored by the group in their later work with a canine whole liver supporting a porcine model of hepatic failure induced by lipopolysaccharide and alpha-amanitin, which demonstrated improvements compared to the porcine group not supported by the liver cross-circulation [149]. The group reported that the lack of immune-mediated reactions was the result of a semipermeable membrane blocking the movement of IgM, which they investigated in later work [150].

Bacchetta et al, who had previously investigated lung cross-circulation also followed up with work on liver cross-circulation in 2022, with a publication using a porcine host for an extracorporeal porcine liver to show improvements in synthetic function, metabolic activity, and histopathological examination [151]. This effort was then replicated using explanted human donor livers that were supported by xenogeneic cross-circulation, which also demonstrated improved histopathologic assessment at 24 h of support [152].

While other organs have not been investigated as extensively, there are reports of cross-circulation being studied in heart transplantation. In one model, standard *ex vivo* cardiac perfusion in a porcine graft was compared to EVHP supplemented with cross-circulation from a paracorporeal support pig [153]. This was then further compared to a third setup in which blood from the support animal was filtered into plasma during cross-circulation. Cross-circulation was reported in both set-ups to have high oxygen consumption and vascular resistance, with the conclusion that it improved preservation compared to standard EVHP. Cross-circulation was also used to support EVHP for 72 h in a study by the Extracorporeal Life Support Laboratory at the University of Michigan, which supported ovine hearts with paracorporeal sheep. All cross-circulated hearts were deemed suitable for transplantation by the end of 72 h, while the control group, which was not cross-circulated, all failed after 6–10 h of EVHP [154]. The group then further explored porcine EVHP support in a publication showing that EVHP could be maintained for 24 h with plasma exchange, which they reported eliminated the need for a paracorporeal animal [155].

## Future Directions in Bioengineering Strategies

The development of bioengineered organs that could be produced in the laboratory would be the ultimate step in alleviating the problems caused by the shortage of donor organs. In the quest to develop such a product, techniques have emerged to grow or build organs. One such method uses organoids made by three-dimensional cell cultures formed from patient-derived cells designed to replicate *in vitro* the microarchitectural and functional characteristics of their *in vivo* counterparts. One advantage of building such models is the ability to further study diseases and potential drugs and therapies on a platform that closely resembles human physiology while reducing the need for animal-based research. In the lung, for example, the advances made in microfluidic organ-on-a-chip devices have been driven by the study of specific disease processes such as those arising from particulate exposure, pulmonary fibrosis, and acute respiratory distress syndrome [156–158].

The study of human organ physiology and disease occurrence through microfluidic devices can be extrapolated for use in transplantation surgery in general. In transplantation, these could represent an avenue for patient-specific study of response to immunosuppression in an effort to optimize regimens, and minimize toxicity, adverse drug reactions, and side effects, balancing these against the risk of rejection [159].

Another potential application is the detection of transplant rejection, which remains a clinical challenge given the high rates of both acute and chronic forms of immune-mediated rejection. Madhvapathy et al. used rat models in which a wireless electronics interface was connected to transplanted kidneys, allowing for real-time monitoring of ultradian rhythms, disruption of the circadian cycle, and organ temperature. These patterns change once immunosuppression is discontinued before creatinine elevation [160].

On top of leveraging advanced techniques to further study transplant-related complications, bioengineering principles could potentially be used to develop artificial organs. In decellularization-recellularization experiments, cellular material is removed during a decellularization process to then be replenished with a new repopulation of cells during the recellularization phase. Decellularization of living tissue can be accomplished by chemical, enzymatic, physical, or combined approaches to generate an extracellular matrix scaffold that provides structural support for the future regenerated organ. The most common materials used for decellularization are a combination of detergents such as sodium dodecyl sulfate (SDS) and Triton-X100 [161]. This is an important step as retaining any cell membrane epitopes would trigger a host response *in vivo.* [162] Once the mechanical integrity has been established and decontaminated, the acellular scaffold undergoes the process of repopulation with organ-specific cells, thus reconstituting the organ from a functional perspective.

For the liver, *in vitro* studies of drug-induced hepatotoxicity, liver function and regenerative potential have advanced the development of primary hepatocyte isolation and the generation of two- and three-dimensional liver models, spheroids, organoids, slice cultures and microfluidic models [163, 164]. Decellularized human liver scaffolds have been repopulated with human cell lines and resulted in liver scaffold cubes that could be successfully xenotransplanted subcutaneously into immunocompetent mice, without any resulting immunogenic responses [165]. Other investigations in animal models have shown that decellularization of the whole organ liver is possible with preservation of the macrostructure [166]. While decellularization has been demonstrated using a variety of methodologies, there is no consensus on the most advantageous approach nor have any unified criteria been accepted [167].

Similarly, studies have reported successful recellularization of kidney scaffolds with preservation of microarchitecture and ECM components such as glycosaminoglycans (GAGs) [168–170]. After preloading of the vascular matrix with vascular endothelial growth factor (VEGF) and angiopoietin 1, pluripotent stem cell-derived endothelial cells can be delivered with efficient adherence, proliferation, and survival [168].

The lung is a challenging organ to bioengineer given the complexity of the cell population that constitutes it. Scaffold generation has been approached both from the angle of using human-derived acellular scaffolds and artificial scaffolds made from synthetic polymers [171–173]. Because there are over 40 different cell types that make up the lung, recellularization is a challenging task and a variety of sources including tissue-isolated progenitor cells, differentiated pluripotent stem cells, and mesenchymal stem cells are being investigated for their potential [174–177].

While these methodologies have a long way to go before they can be considered for clinical application, the progress being made is promising for the advent of engineered organs to meet the demand for transplantation.

## Conclusion

As the definitive management for end-stage organ disease, transplantation remains an in-demand procedure. While surgical and perioperative techniques have progressed, there is still a notable shortcoming, notably the mismatch between the number of available organs and the need for grafts by potential recipients on the waiting list. In order to bridge the gap between supply and demand, new methods are needed for the recuperation of suitable organs. Machine perfusion provides an emerging platform for the recovery and regeneration of organs that would otherwise be discarded. Advancements made in gene therapy, applications of mesenchymal stem cells, cross-circulation methodologies, and the advent of advanced bioengineering principles have been covered in this review as promising avenues for organ recovery. While many of these approaches are in various stages of development and clinical application, they represent important advances that can be leveraged in the search for meaningful ways to increase the donor pool.
